# miR-197-3p Represses the Proliferation of Prostate Cancer by Regulating the VDAC1/AKT/β-catenin Signaling Axis

**DOI:** 10.7150/ijbs.42019

**Published:** 2020-02-21

**Authors:** Qiang Huang, Bo Ma, Yixi Su, Kawo Chan, Hu Qu, Jiayu Huang, Dejuan Wang, Jianguang Qiu, Huanliang Liu, Xiangling Yang, Zhongyang Wang

**Affiliations:** 1 Department of Urology, The Sixth Affiliated Hospital, Sun Yat-sen University, Guangzhou, Guangdong 510655, China; 2 Guangdong Provincial Key Laboratory of Colorectal and Pelvic Floor Diseases, Guangdong Institute of Gastroenterology, The Sixth Affiliated Hospital, Sun Yat-sen University, Guangzhou, Guangdong 510655, China; 3 Department of Clinical Laboratory, The Sixth Affiliated Hospital, Sun Yat-sen University, Guangzhou, Guangdong 510655, China

**Keywords:** miR-197-3p, prostate cancer, VDAC1, AKT

## Abstract

Accumulating investigations have demonstrated that microRNAs (miRNAs) are promising efficient targets for the next generation of molecular therapeutics. The development of miRNA-based therapies requires the identification and validation of cancer-associated miRNAs. Herein, we identified that miR-197-3p regulates the carcinogenesis and development of prostate cancer (PCa) via bioinformatics analysis. Next, we investigated the function and regulatory mechanisms of miR-197-3p in PCa. Overexpression of miR-197-3p suppressed PCa cell proliferation and colony formation. In contrast, inhibition of miR-197-3p activity enhanced PCa cell proliferation and colony formation. Mechanistic investigations identified that voltage dependent anion channel 1 (VDAC1) is a direct target of miR-197-3p. miR-197-3p targeting of VDAC1 resulted in downregulation of p-Akt and β-catenin. Subsequently, we found that restoration of VDAC1 abolished the effects of miR-197-3p on PCa cell proliferation and AKT signaling pathway. Furthermore, we confirmed that miR-197-3p suppressed tumor xenograft growth in *vivo*. In conclusion, our study offers an empirical investigation of miR-197-3p, a tumor suppressor that may be a potential therapeutic target in PCa.

## Introduction

Prostate cancer (PCa) is the second most prevalent cancer for males worldwide, and the number of PCa cases in 2018 was nearly 1.27 million 1. In general, androgen deprivation therapy (ADT) benefits PCa patients in the early stage. However, progression from the androgen-sensitive stage to castration-resistant prostate cancer (CRPC) is inevitable 2-3. There are few available treatment options for patients suffering from CRPC. Although the application of enzalutamide and abiraterone improves the overall survival of patients, the effect is short-lived, and the development of drug resistance leads to the failure of treatment 4-5. Identification of novel therapeutic targets for prostate cancer treatment is urgent.

Emerging evidence has indicated that microRNAs (miRNAs) have potential for the diagnosis and treatment of prostate cancer 6. miRNAs are short noncoding RNAs consisting of 18-22 nucleotides, which bind to the 3′-untranslated region (3′-UTR) of target mRNAs to breakdown mRNA or repress translation 7. Previous investigations have shown that miRNAs play significant roles in cell cycle, differentiation, apoptosis and tumorigenesis 8-9. The dysfunction of miRNAs may promote or suppress the progression of tumors, such as prostate cancer 10. Aberrant expression of 50 miRNAs has been observed in prostate cancer, but only a few were experimentally proven to contribute to the disease 11.

miR-197-3p, as an oncogenic miRNA, has been reported to promote cell proliferation, migration, and invasion in bladder cancer cells 12. Moreover, LIFR-AS1 inhibits breast cancer cell proliferation and migration by binding to miR-197-3p 13. In lung cancer cells, the miR-197-3p/p120 catenin axis is regulated by lncRNA MALAT1 to depress chemo-sensitivity 14. miR-197-3p has also been confirmed to promote the invasion and migration of thyroid cancer cells 15. However, in hepatocellular carcinoma, miR-197-3p inhibits cell invasion as a favorable prognosis marker 16. Overall, miR-197-3p performs different roles in multiple malignancies. For prostate cancer, only one investigation has demonstrated that miR-197-3p can be combined with other miRNAs to diagnose prostate cancer 17. These findings indicate that miR-197-3p may have particular effects on the development and progression of prostate cancer.

Herein, our study revealed that miR-197-3p suppressed proliferation and colony formation of prostate cancer cells (C4-2 and DU145). Moreover, cell cycle was induced to arrest at G0/G1 phase. Further investigation demonstrated that VDAC1 is a direct target of miR-197-3p via dual-luciferase report assay. Specifically, we hypothesized that the VDAC1/AKT/β-catenin signaling axis is involved in the suppression of PCa cell proliferation resulting from miR-197-3p. These experimental findings suggested that miR-197-3p may be beneficial for PCa-targeted therapy.

## Materials and Methods

### Cell culture and transfection

Human PCa cell lines ( C4-2, DU145 and 22Rv1) were kind gifts from Dr. Jianguang Qiu (Sun Yat-sen University). PCa cell lines were cultured in RPMI-1640 medium (Gibco, USA) supplemented with 10% fetal bovine serum (FBS, Gibco, USA). All cell lines were maintained in a 5% CO_2_ and 37℃ humidified incubator. For cell transfection, cells were cultured in 6-well plates for 24 h. miR-197-3p mimics, mimics negative control, miR-197-3p inhibitor and inhibitor negative control (20 nM, GenePharma, China) were transfected into cells using Lipofectamine® RNAiMAX (Invitrogen, USA). Transfection efficiency was detected after 48 h. The RNA oligo sequences are shown in Table S1.

### Dual luciferase reporter assay

To determine if the 3'-UTR of VDAC1 interacts with miR-197-3p, a dual luciferase reporter assay was performed as previously reported 18. Briefly, 1×10^5^ C4-2 and DU145 cells were seeded in 24-well plates. After 24 h, miR-197-3p mimics and negative control together with VDAC1 WT and MUT plasmids (Vigene Biosciences, USA) were transfected into cells using Lipofectamine 3000 (Invitrogen, USA). Luciferase activities were then analyzed.

### Western blot and antibodies

Radio-immunoprecipitation assay (RIPA; Beyotime, China) lysis buffer was used to obtain proteins from C4-2 and DU145 cells. We measured protein concentration by the bicinchoninic acid (BCA) assay. After the SDS-polyacrylamide gel electrophoresis assay and transfer to PVDF membrane (Immobilon-P Membrane, USA), membranes were blocked by 5% skim milk (BD Biosciences, USA) and then, incubated with primary antibodies at 4℃ overnight. Subsequently, membranes were incubated with HRP-conjugated rabbit or mouse secondary antibodies (Thermo Scientific, USA) for 1 h, and a chemiluminescence reagent was used for development, exposure and analysis of protein bands. The following primary antibodies were used: anti-p53 (104421-1-AP), p21 (2947, CST), CyclinD1 (2978, CST), VDAC1 (55259-1-AP, Proteintech), FOXO3 (12829S, CST), p-AKT (4058S, CST), AKT (4694S, CST), β-catenin (ab32572, Abcam) and GAPDH (10494-1-AP, Proteintech).

### EdU assay

An EdU assay was performed using an EdU assay kit (RiboBio, China). Cells were plated into 96-well plates (2×10^4^ cells/well), incubated with EdU (50 µM) for 2 h and fixed in 4% formaldehyde (Leagene, China) for 30 min. Subsequently, cells were permeabilized with 0.3% TritonX-100 (Jiayan Biotech, China) for 10 min and then incubated with glycine (2 mg/mL, Sangon Biotech, China) for 5 min. Cells were reacted with Apollo reaction mixture for 30 min. Cell nuclei were stained with Hoechst 33342 (Invitrogen, USA) for 30 min and visualized under a microscope.

### Cell proliferation and colony formation assay

Cell proliferation assay (5×10^3^ cells/well) was performed on the xCELLigence system (ACEA Biosciences, USA) as previously described 19. For colony formation assay, 1×10^3^ Cells were plated into 6-well plates. After 9 days, complete medium was removed, cells were washed twice with PBS and fixed in 4% formaldehyde for 20 min. Subsequently, cells were stained with Coomassie brilliant blue (Beyotime, China) for 20 min, and colony number was then counted.

### Quantitative real-time PCR (qRT-PCR)

Total RNA was isolated using the RNA-Quick Purification kit (Yishan Biotechnology, China). For miRNA, reverse transcription was performed using the PrimeScript™ RT reagent kit (Takara, Japan). Real-Time PCR was performed utilizing an ABI ViiA7 fast real-time PCR system and SYBR Green gene expression assays according to the standard protocol. For VDAC1 mRNA expression, reverse transcription was performed using the PrimeScript™ RT reagent kit with gDNA Eraser (Takara, Japan). After qPCR, relative gene expression was analyzed by the -△△Ct method. PCR primer sequences are presented in Table S2.

### Cell cycle analysis

Cells were resuspended in 1 ml of PBS and then fixed with 3 ml of anhydrous ethanol. After incubation at 4 °C for 1 h, 500 µL of PBS was used to replace the anhydrous ethanol. After incubation with 500 µL of RNaseA (BD Pharmingen, USA) for 20 min, cell cycle was evaluated by flow cytometry.

### Immunofluorescent staining

After washing with PBS, cells were fixed in 4% formaldehyde for 15 min and permeabilized with 0.3% TritonX-100 for 10 min, cells were blocked with 10% goat serum (BosterBio, USA) for 1 h and then incubated in primary antibody diluted with 2% goat serum at 4 °C overnight. After incubation with secondary antibodies and visualization of nuclei with DAPI (Beyotime, China), images were processed and analyzed using a confocal laser scanning microscope (Leica, Germany).

### Immunohistochemistry

Paraffin-embedded tissues were deparaffinized with xylene and alcohol. Endogenous peroxidase activity was blocked with 0.3% H_2_O_2_ for 10 min and an antigen retrieval process was performed using high pressure with tissues incubated in ethylene diamine tetraacetic acid (EDTA) buffer. Subsequently, slides were incubated at 4 °C overnight with primary antibodies, and slides were then incubated with secondary antibodies for 1 h. After counterstaining with hematoxylin, slides were prepared for microscopic evaluation.

### Tumor xenograft model

To further confirm the effect of miR-197-3p on PCa cells in *vivo*, 4×10^6^ C4-2 cells were subcutaneously injecting into the flanks of 4-week-old male BALB/c-nu mice (Nanjing Biomedical Research Institute of Nanjing University). A mixture of miR-197-3p mimics and transfected liposomes (Engreen Biosystem, China) were injected into the surrounding area of the tumor (every 3 days). Tumor volume was measured using a standard caliper, and it was calculated with the following formula: V=L×W^2^/2. All mice were sacrificed using euthanasia after 4 weeks, and xenograft tumors were collected for immunohistochemistry and qPCR. All animal experiments were performed according to standard protocols approved by Animal Ethical and Welfare Committee of Sun Yat-sen University (20190718-003).

### Statistical analysis

All data obtained were analyzed by GraphPad Prism 8.0 software. Results are presented as the mean ± standard deviation (SD). One-way analysis of variance (ANOVA) followed by Tukey's post hoc multiple-comparison tests was applied to analyze significant differences when more than two groups were compared. T-test was used to compare two independent groups. *P* < 0.05 was considered to be statistically significant. Samples were independent biological replicates.

## Results

### Preliminary screening of miRNAs associated with PCa

**Figure 1:**
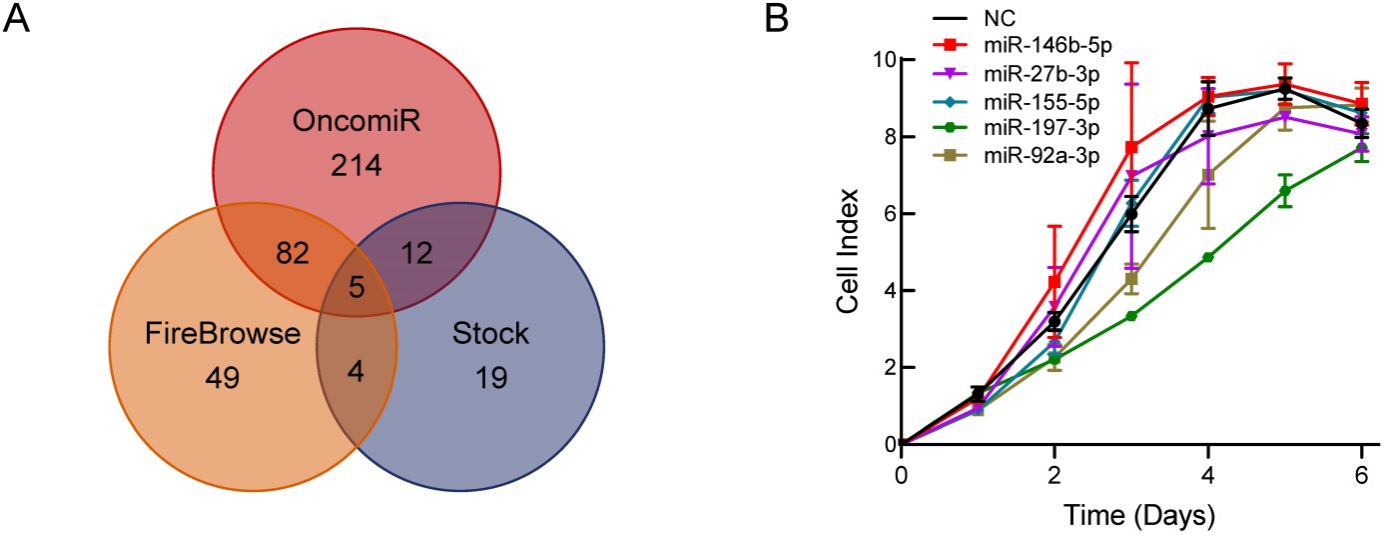
** Bioinformatics analysis and experimental screening identify miRNAs that affect PCa cell growth. (A)** Venn diagrams show the intersections of miRNAs associated with PCa. **(B)** Cell proliferation screening of predicted miRNAs.

To explore a novel small molecule that may affect PCa cell growth, we utilized the OncomiR and FireBrowse websites to screen for potential miRNAs associated with PCa. The OncomiR website provided 313 miRNAs that correlated with PCa progression and development and then 140 differential expressed miRNAs in PCa were obtained from the FireBrowse website. Combined with the 40 miRNAs related to serum starvation collected in our laboratory, we generated Venn diagrams to illustrate the intersections of miRNAs associated with PCa (Figure 1A). Five miRNAs were selected for further functional investigations. miR-197-3p was the most potent miRNA that inhibited the growth of C4-2 cells (Figure 1B).

### miR-197-3p suppresses the proliferation and colony formation of PCa cells

**Figure 2:**
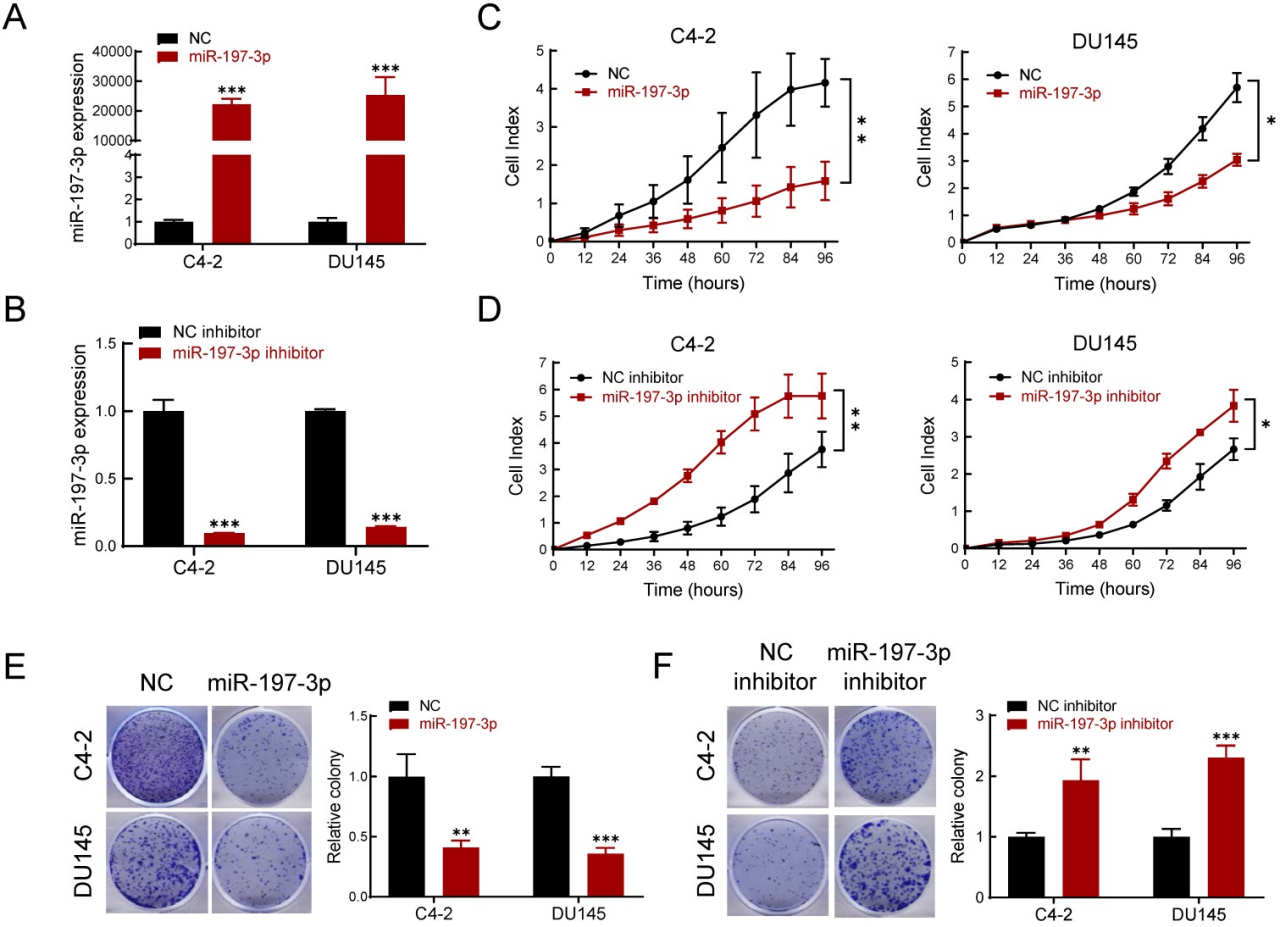
** miR-197-3p inhibits cell proliferation and colony formation in PCa cells. (A-B)** qPCR was used to verify the relative expression of miR-197-3p in C4-2 and DU145 cells transfected with miR-197-3p mimics, negative control, miR-197-3p inhibitor or inhibitor negative control. **(C-D)** RTCA assay was performed to evaluate the effect of miR-197-3p overexpression or downregulation on PCa cell proliferation. **(E-F)** Colony formation results of PCa cells transfected with mimics or inhibitor. (Data are represented as the mean ± SD; *P < 0.05, **P < 0.01, ***P < 0.001).

To determine the effect of miR-197-3p on PCa cell phenotypes, transfection of miR-197-3p mimics was used to up-regulate miR-197-3p expression (Figure 2A), and a miR-197-3p inhibitor was used to down-regulate miR-197-3p expression (Figure 2B). The Real-time cell analysis (RTCA) showed that overexpression of miR-197-3p suppressed the proliferation of C4-2 and DU145 cells compared to the negative control (Figure 2C). Moreover, inhibition of miR-197-3p facilitated cell proliferation (Figure 2D). After 10 days of incubation, the colony formation assay showed that overexpression of miR-197-3p resulted in fewer and smaller colonies compared to negative controls in C4-2, DU145 and 22Rv1 cells (Figure 2E and Figure S1). In agreement, inhibition of miR-197-3p resulted in the opposite effect according to the colony formation assay (Figure 2F).

### miR-197-3p reduces DNA replication and arrests cell cycle

**Figure 3:**
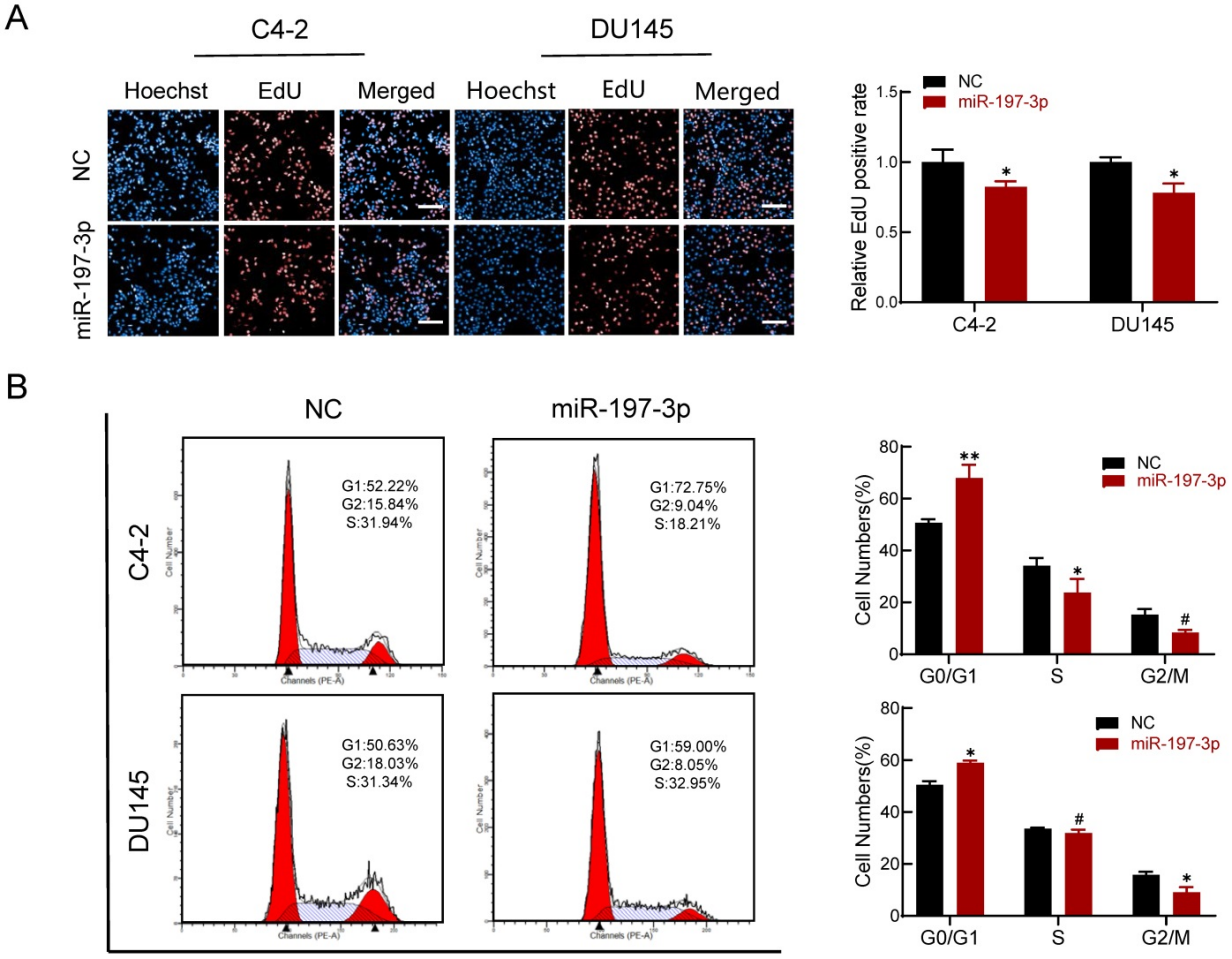
** miR-197-3p reduces DNA replication and arrests cell cycle in PCa cells. (A)** Effect of miR-197-3p on DNA replication of PCa cells according to the EdU assay. Scale bar=20 µm. **(B)** The cell cycle of PCa cells transfected with miR-197-3p mimics or negative control was measured in flow cytometry analysis. (Data are represented as the mean ± SD; *P < 0.05, **P < 0.01, #: no significance).

An EdU assay was performed to investigate if miR-197-3p is involved in DNA replication. Overexpression of miR-197-3p inhibited DNA replication (Figure 3A), verifying that miR-197-3p suppresses cell proliferation. We next evaluated the role of miR-197-3p in the cell cycle. Flow cytometry analysis revealed that overexpression of miR-197-3p blocked cell cycle progression at the G0/G1 phase (Figure 3B). In addition, Western blotting showed that miR-197-3p up-regulated the expression of p53 and p21 cell cycle-related proteins (Figure 5A). These results indicated that miR-197-3p blocks cell cycle progression at the G0/G1 phase by inhibiting DNA replication.

### Downstream expression profiling of miR-197-3p overexpression

**Figure 4:**
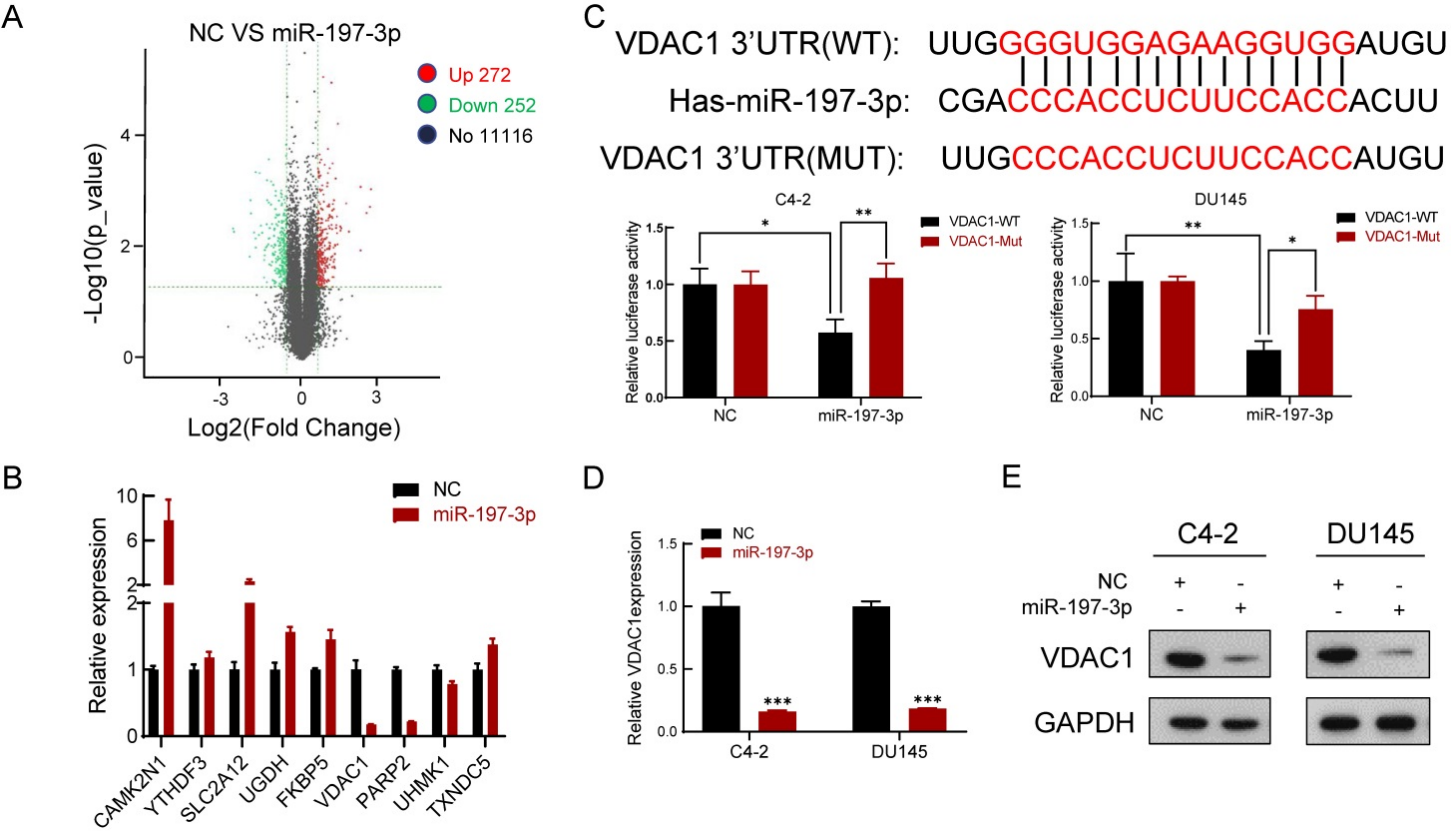
** VDAC1 is a direct target of miR-197-3p in PCa cells. (A)** The gene expression profile of C4-2 cells with miR-197-3p overexpression. **(B)** qPCR analysis of the expression of significant differential genes. **(C)** miR-197-3p and its putative binding sequence of VDAC1 or its mutated form were inserted into the plasmid vector. Luciferase reporter constructs containing wild-type or mutated VDAC1 3′ UTRs were co-transfected with miR-197-3p mimics or negative control into PCa cells. Luciferase activities were examined, and relative firefly luciferase expression was normalized to Renilla luciferase. **(D-E)** qPCR and western blot analysis of VDAC1 expression after transfection of miR-197-3p and negative control in PCa cells. (Data are represented as the mean ± SD; *P < 0.05, **P < 0.01, ***P < 0.001).

We next performed gene expression profiling of miR-197-3p overexpression in C4-2 cells. The analysis identified 272 and 252 genes that were induced and inhibited, respectively (Figure 4A). We then verified five upregulated genes and four downregulated genes by qPCR (Figure 4B), which was consistent with the sequencing results. VDAC1 was one of the most downregulated genes in miR-197-3p-overexpressing cells.

### miR-197-3p directly targets VDAC1 and affects the AKT signaling pathway

**Figure 5:**
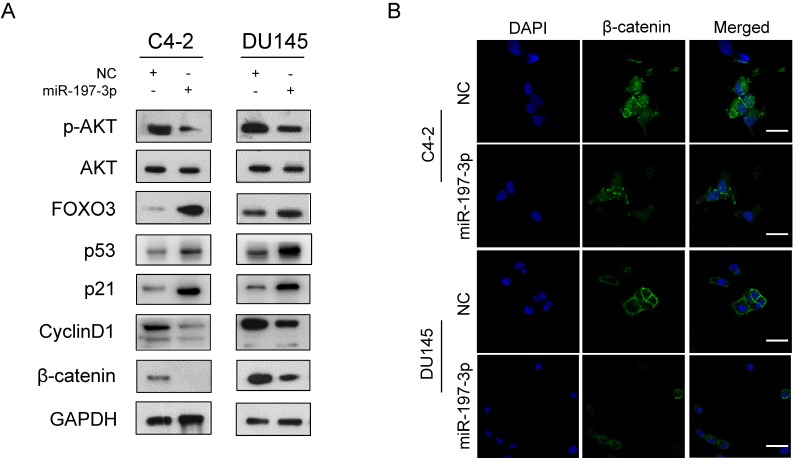
** miR-197-3p affects the AKT signaling pathway. (A)** Protein levels associated with the AKT signaling pathway and cell cycle.** (B)** Representative immunofluorescence images of β-catenin expression (green signal) in PCa cells transfected with NC or miR-197-3p mimics for 48 h. DAPI (blue signal) was used to counterstain cell nuclei. Scale bar=40 µm.

To investigate the underlying mechanism of miR-197-3p in PCa cell proliferation, two websites (TargetScan and miRWalk) were used to predict the biological target of miR-197-3p. Combined with transcriptome sequencing results, we determined that VDAC1 may be a potential target of miR-197-3p. Dual-luciferase reporter analysis showed that the expression of miR-197-3p significantly inhibited the activity of firefly luciferase with the wild-type, but not mutant, 3'UTR of VDAC1 (Figure 4C). Furthermore, we detected lower expression of VDAC1 at protein and mRNA levels after transfection of miR-197-3p mimics (Figure 4D&E). Therefore, these results indicated that miR-197-3p directly targets VDAC1 by binding to the 3'UTR of VDAC1. Previous research has reported that VDAC1 is associated with the AKT signaling pathway 20. In order to investigate the potential mechanism by which miR-197-3p affects AKT signaling pathway, downstream proteins of AKT signaling pathway were detected. Overexpression of miR-197-3p inhibited the expression of p-Akt, β-catenin and CyclinD1, but promoted the expression of FOXO3 (Figure 5A). Immunofluorescent staining also confirmed that the expression of β-catenin was inhibited in PCa cells transfected with miR-197-3p mimics (Figure 5B). These results suggested that miR-197-3p directly targets VDAC1 and regulates the AKT signaling pathway.

### VDAC1 recovers the effect of miR-197-3p on PCa cell proliferation

**Figure 6:**
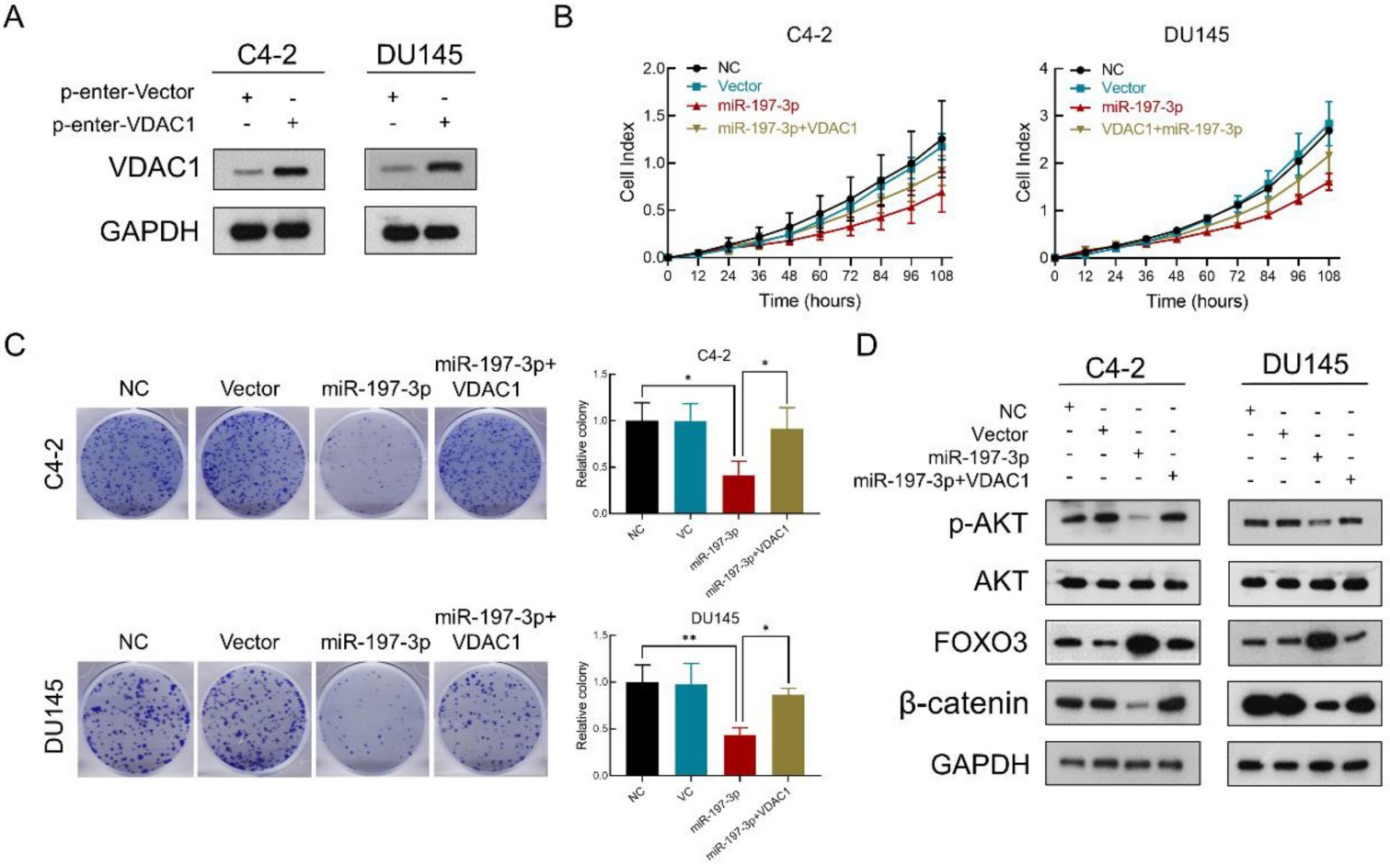
** VDAC1 recovers the effect of miR-197-3p on PCa cell proliferation. (A)** VDAC1 overexpression plasmid was constructed and then tested for transfection efficiency. **(B)** RTCA results of PCa cells transfected with miR-197-3p mimics or co-transfected with miR-197-3p mimics and VDAC1 plasmid. **(C)** Colony formation results of PCa cells transfected with miR-197-3p mimics or co-transfected with miR-197-3p mimics and VDAC1 plasmid. **(D)** Western blot analysis indicated that VDAC1 recovered the effect of miR-197-3p on AKT signaling pathway proteins. (Data are represented as the mean ± SD; *P < 0.05, **P < 0.01).

To verify that miR-197-3p suppresses PCa cell proliferation by downregulating VDAC1, we constructed a VDAC1 overexpression plasmid. Transfection efficiency was detected after transfection of this plasmid into C4-2 and DU145 cells. Compared to the p-Enter-VC group, the p-Enter-VDAC1 group achieved high-expression level of VDAC1 (Figure 6A). Colony formation and RTCA assays were used to evaluate cell proliferation after transfection. The results showed miR-197-3p suppressed C4-2 and DU145 cells growth. Moreover, VDAC1 contributed to the recovery of cell growth inhibition (Figure 6B&C). To further investigate if miR-197-3p affects the AKT signaling pathway via inhibiting VDAC1, we transfected cells with the VDAC1 plasmid, VDAC1 plasmid+miR-197-3p mimics or vector control. Western blotting revealed that VDAC1 reversed the changes induced by miR-197-3p, such as downregulation of p-Akt and β-catenin as well as upregulation of FOXO3 (Figure 6D). These data further identified that miR-197-3p suppresses PCa cell growth via the VDAC1/AKT/β-catenin signaling axis.

### miR-197-3p suppresses xenograft tumor growth in BALB/c-nu mice

**Figure 7:**
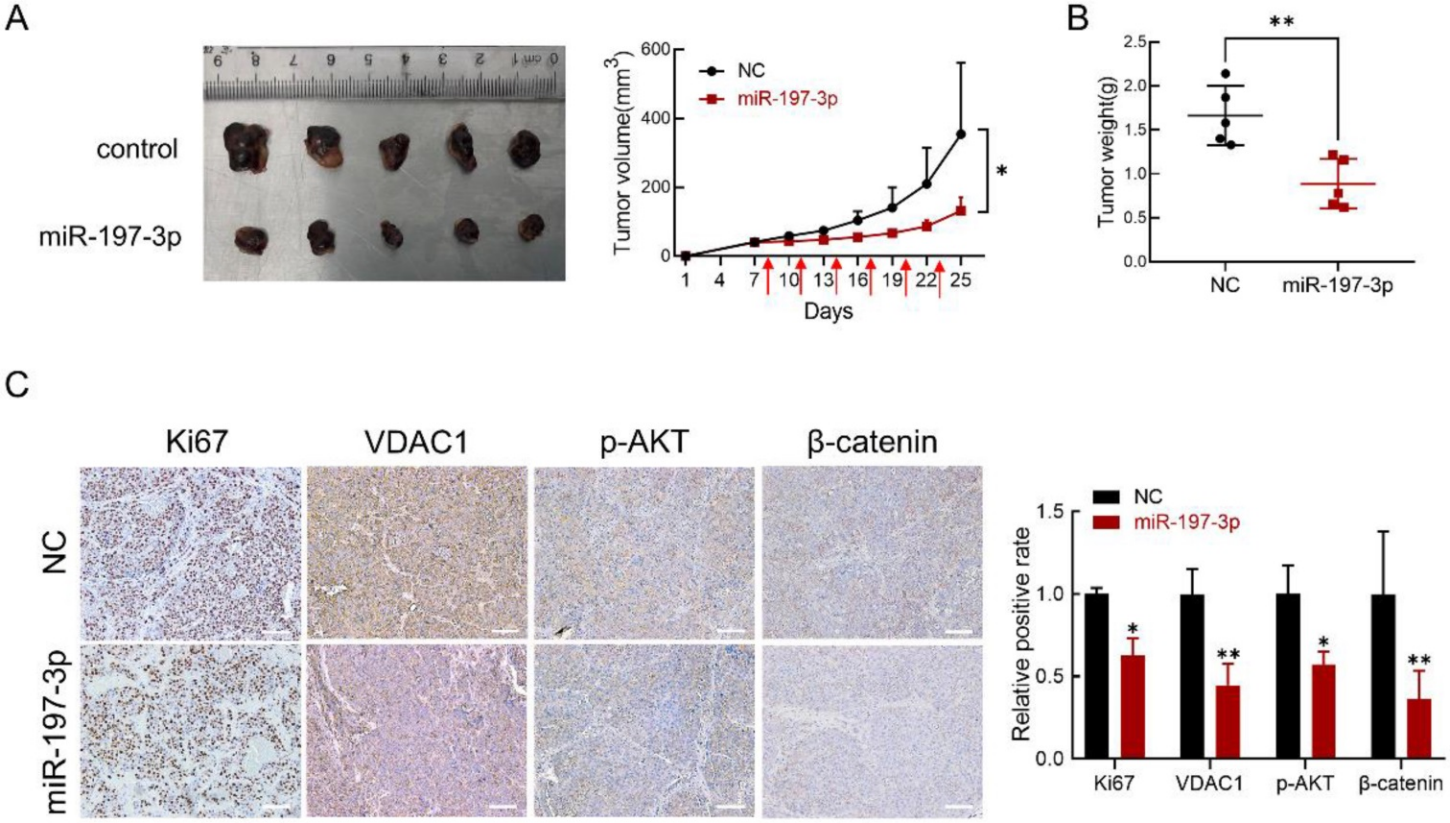
** miR-197-3p suppresses PCa tumor growth in *vivo*. (A)** Tumors were obtained from nude mice injected subcutaneously with 3×10^6^ C4-2 cells transfected with miR-197-3p mimics or negative control, respectively. Tumor volume was measured every 3 days. The time of administration is indicated with red arrows. **(B)** Measurement of xenograft tumor weight. **(C)** Immunohistochemical staining for Ki67, VDAC1, p-AKT and β-catenin. Scale bar=100 µm. (Data are represented as the mean ± SD; *P < 0.05, **P < 0.01).

BALB/c-nu mice were used to investigate the effect of miR-197-3p in *vivo*. We subcutaneously inoculated C4-2 cells to establish a xenograft model. After 8 days, miR-197-3p mimics or mimics negative control were injected into the surrounding area of the tumor every 3 days. miR-197-3p mimics-based treatment significantly reduced tumor weight and tumor volume (Figure 7A&B). Immunohistochemistry was performed to validate the effect of miR-197-3p mimics on tumor proliferation ability and the expression of Ki67, VDAC1, p-AKT and β-catenin. The expression of Ki67, VDAC1, p-AKT and β-catenin was low in tumors treated with miR-197-3p mimics (Figure 7C). qRT-PCR was performed to confirm the treatment efficiency of miR-197-3p mimics. Compared to the NC group, the miR-197-3p group expressed higher level of miR-197-3p (Figure S3). These results indicated that miR-197-3p suppresses xenograft tumor growth in BALB/c-nu mice.

## Discussion

MiRNAs regulate nearly 30% of human genes and have a significant role in the pathogenesis of various cancers 21. Aberrant expression of miRNAs in cancers indicates a potential therapeutic direction 22-23. After determining the role of miRNAs in pathogenesis and downstream targets, we can utilize mimics or antagonistic small nucleic acids to suppress cancers. Increasing numbers of preclinical studies have focused on the regulation of miRNAs in prostate cancer. It has been reported that miR-15a and miR-16-1 down-regulate BCL2, CCND1 and WNT3A, resulting in inhibition of PCa cell survival and invasion 24. In addition, overexpression of miR-34a inhibits subcutaneous tumor growth and reduces lung metastasis in an orthotopic tumor model 25. However, miRNA-based therapies have not been advanced into clinical trials in PCa 26. The main challenge is the selection of potent miRNA candidates and targeted delivery system. Thus, it is essential to explore more novel PCa-related miRNAs and gene targets.

In this study, we screened five miRNAs potentially associated with PCa via bioinformatics prediction. A RTCA assay was performed to clarify the effect on C4-2 cell proliferation. Interestingly, only miR-197-3p significantly inhibited cell proliferation. Accumulating studies have demonstrated that miR-197-3p plays different roles in many tumors. However, the underlying mechanism of how miR-197-3p affects PCa progression remains unclear. We demonstrated that miR-197-3p suppressed cell proliferation and colony formation of C4-2 and DU145 cells. When the miR-197-3p inhibitor was transfected into C4-2 and DU145 cells, the results showed the opposite effect. We next performed Transwell assays and invasion assays to determine the effect of miR-197-3p on PCa cell migration and invasion, but the results showed no significant difference (Figure S2). EdU proliferation assays indicated that miR-197-3p reduced DNA replication in both C4-2 and DU145 cells. Flow cytometry results showed that miR-197-3p blocked the cell cycle at G0/G1 phase. Ample findings have revealed that DNA damage usually activates the p53-p21 pathway and causes G1 phase arrest in mammalian cells 27-28. Therefore, we tested the protein levels of p53 and p21. As expected, overexpression of miR-197-3p may regulate the p53-p21 pathway, thereby leading to G1 phase arrest. Taken together, these data indicated that miR-197-3p acts as a tumor suppressor in the negative regulation of PCa cell proliferation but without affecting migration and invasion.

Voltage dependent anion channel 1 (VDAC1) is a major component of the outer mitochondrial membrane, contributing to metabolite and ion exchange across the outer mitochondrial membrane and possibly regulating mitochondrial functions 29. VDAC1 has been shown to participate in tumor progression in various cancers. Inhibition of VDAC1 expression by siRNA suppresses cell proliferation and tumor growth in cancers, including lung, prostate, colon, glioblastoma, liver and pancreas cancer 30. Mechanistically, VDAC1 directly interacts with Mcl-1 to regulate the generation of ROS in lung cancer 31. VDAC1 also plays a significant role in mitochondria-mediated apoptosis and targets to modulate apoptosis 32. In this study, two miRNA target prediction websites (TargetScan and miRWalk) were combined with mRNA transcriptome sequencing results to explore the most potential target gene of miR-197-3p. Furthermore, VDAC1 was verified to be a target of miR-197-3p in PCa cells by dual luciferase reporter assays. Subsequently, we confirmed that miR-197-3p regulated the downstream AKT signaling pathway, which is widely involved in cell proliferation, differentiation, apoptosis and migration. Moreover, restoration of VDAC1 abolished the effects of miR-197-3p on PCa cell proliferation and AKT signaling pathway. Thus, our findings indicated that miR-197-3p inhibits PCa cell growth via targeting VDAC1 and regulating AKT signaling pathway.

## Conclusions

**Figure 8:**
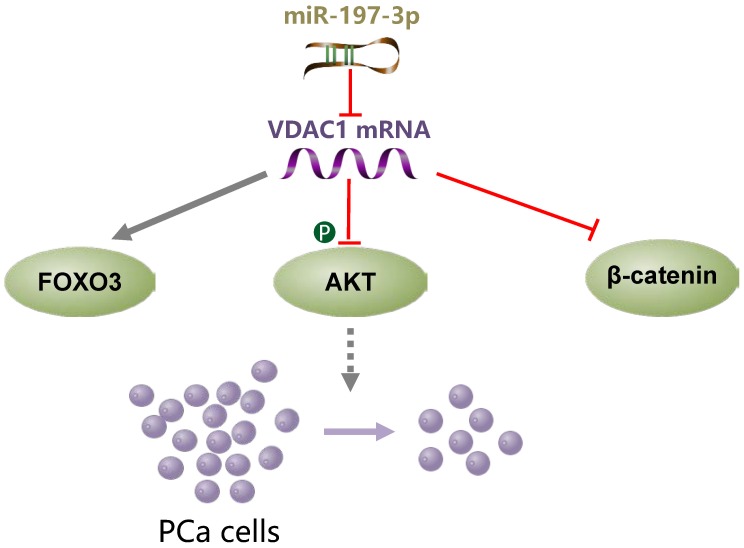
A schematic model of the mechanism indicating how miR-197-3p suppresses prostate cancer cell proliferation.

miR-197-3p overexpression suppresses cell proliferation without affecting migration and invasion in *vitro* and in *vivo*. VDAC1 was identified as a direct target gene of miR-197-3p. Further investigation of the underlying mechanism demonstrated that the miR-197-3p/VDAC1/AKT/β-catenin signaling axis regulates PCa cell growth (Figure 8). Additional potential molecular pathways and potent drug delivery systems need to be explored to enhance the feasibility of miRNA-based therapy in PCa.

## Supplementary Material

Supplementary figures and tables.

## References

[1] Bray F, Ferlay J, Soerjomataram I (2018). Global cancer statistics 2018: GLOBOCAN estimates of incidence and mortality worldwide for 36 cancers in 185 countries. A Cancer Journal for Clinicians.

[2] Wang Q, Li W, Zhang Y (2009). Androgen Receptor Regulates a Distinct Transcription Program in Androgen-Independent Prostate Cancer. Cell.

[3] Pomerantz MM, Li F, Takeda DY (2015). The androgen receptor cistrome is extensively reprogrammed in human prostate tumorigenesis. Nature Genetics.

[4] van Soest RJ, van Royen ME, de Morrée ES (2013). Cross-resistance between taxanes and new hormonal agents abiraterone and enzalutamide may affect drug sequence choices in metastatic castration-resistant prostate cancer. European Journal of Cancer.

[5] Annala M, Vandekerkhove G, Khalaf D (2018). Circulating Tumor DNA Genomics Correlate with Resistance to Abiraterone and Enzalutamide in Prostate Cancer. Cancer Discovery.

[6] Stegeman S, Amankwah E, Klein K (2015). A Large-Scale Analysis of Genetic Variants within Putative miRNA Binding Sites in Prostate Cancer. Cancer Discovery.

[7] Bartel DP (2009). MicroRNAs: Target Recognition and Regulatory Functions. Cell.

[8] Brennecke J, Hipfner DR, Stark A (2003). bantam Encodes a Developmentally Regulated microRNA that Controls Cell Proliferation and Regulates the Proapoptotic Gene hid in Drosophila. Cell.

[9] Ivey KN Srivastava D MicroRNAs as Regulators of Differentiation and Cell Fate Decisions Cell Stem Cell 2010 7041 10.1016/j.stem.2010.06.012 20621048

[10] Lin S, Gregory RI (2015). MicroRNA biogenesis pathways in cancer. Nature Reviews Cancer.

[11] Catto JWF, Alcaraz A, Bjartell AS (2011). MicroRNA in Prostate, Bladder, and Kidney Cancer: A Systematic Review. European Urology.

[12] Jiang Y, Wei T, Li W (2019). Circular RNA hsa_circ_0002024 suppresses cell proliferation, migration, and invasion in bladder cancer by sponging miR-197-3p. American Journal of Translational Research.

[13] Xu F, Li H, Hu C (2019). LIFR-AS1 modulates Sufu to inhibit cell proliferation and migration by miR-197-3p in breast cancer. Bioscience Reports.

[14] Yang T, Li H, Chen T (2019). LncRNA MALAT1 Depressed Chemo-Sensitivity of NSCLC Cells through Directly Functioning on miR-197-3p/p120 Catenin Axis. Molecules and Cells.

[15] Liu K, Huang W, Yan D (2017). Overexpression of long intergenic noncoding RNA LINC00312 inhibits the invasion and migration of thyroid cancer cells by down-regulating microRNA-197-3p. Bioscience Reports.

[16] Ni J, Zheng H, Huang Z (2019). MicroRNA-197-3p acts as a prognostic marker and inhibits cell invasion in hepatocellular carcinoma. Oncology Letters.

[17] Daniel R, Wu Q, Williams V (2017). A Panel of MicroRNAs as Diagnostic Biomarkers for the Identification of Prostate Cancer. International Journal of Molecular Sciences.

[18] Yang X, Du WW, Li H (2013). Both mature miR-17-5p and passenger strand miR-17-3p target TIMP3 and induce prostate tumor growth and invasion. Nucleic Acids Research.

[19] Wen C, Chen J, Zhang D (2016). Pseudolaric acid B induces mitotic arrest and apoptosis in both 5-fluorouracil-sensitive and -resistant colorectal cancer cells. Cancer Letters.

[20] Li L, Yao Y, Gu X (2014). Plasminogen Kringle 5 Induces Endothelial Cell Apoptosis by Triggering a Voltage-dependent Anion Channel 1 (VDAC1) Positive Feedback Loop. Journal of Biological Chemistry.

[21] Guil S, Esteller M (2009). DNA methylomes, histone codes and miRNAs: Tying it all together. The International Journal of Biochemistry & Cell Biology.

[22] Garzon R, Calin GA, Croce CM (2009). MicroRNAs in Cancer. Annual Review of Medicine.

[23] Shenouda SK, Alahari SK (2009). MicroRNA function in cancer: oncogene or a tumor suppressor?. Cancer and Metastasis Reviews.

[24] Bonci D, Coppola V, Musumeci M (2008). The miR-15a-miR-16-1 cluster controls prostate cancer by targeting multiple oncogenic activities. Nature Medicine.

[25] Liu C, Kelnar K, Liu B (2011). The microRNA miR-34a inhibits prostate cancer stem cells and metastasis by directly repressing CD44. Nature Medicine.

[26] Gordanpour A, Nam R, Sugar L (2012). MicroRNAs in prostate cancer: from biomarkers to molecularly-based therapeutics. Prostate Cancer Prostatic Diseases.

[27] He G, Siddik ZH, Huang Z (2005). Induction of p21 by p53 following DNA damage inhibits both Cdk4 and Cdk2 activities. Oncogene.

[28] Seoane J, Le HV, Massagué J (2002). Myc suppression of the p21Cip1 Cdk inhibitor influences the outcome of the p53 response to DNA damage. Nature.

[29] Tomasello F, Messina A, Lartigue L (2009). Outer membrane VDAC1 controls permeability transition of the inner mitochondrial membrane in cellulo during stress-induced apoptosis. Cell Research.

[30] Arif T, Vasilkovsky L, Refaely Y (2014). Silencing VDAC1 Expression by siRNA Inhibits Cancer Cell Proliferation and Tumor Growth In *Vivo*. Molecular Therapy-Nucleic Acids.

[31] Huang H, Shah K, Bradbury NA (2014). Mcl-1 promotes lung cancer cell migration by directly interacting with VDAC to increase mitochondrial Ca2+ uptake and reactive oxygen species generation. Cell Death Disease.

[32] Barmatz V, Kelin Y, Chen Q (2017). VDAC1 as a Player in Mitochondria-Mediated Apoptosis and Target for Modulating Apoptosis. Current Medicinal Chemistry.

