# Supplementary material for: miR-197-3p Represses the Proliferation of Prostate Cancer by Regulating the VDAC1/AKT/β-catenin Signaling Axis
Source: Int J Biol Sci. 2020 Feb 21;16(8):1417–26. doi: 10.7150/ijbs.42019 (PMC7085225; doi:10.7150/ijbs.42019)

Supplementary Tables and Figures

Table S1. RNA oligo sequences.

RNA oligo		Sequence
Negative control	5’ to 3’	UUCUCCGAACGUGUCACGUTT
		ACGUGACACGUUCGGAGAATT
Hsa-miR-146b-5p mimics	5’ to 3’	UGAGAACUGAAUUCCAUAGGCUG
		GCCUAUGGAAUUCAGUUCUCAUU
Hsa-miR-27b-3p mimics	5’ to 3’	UUCACAGUGGCUAAGUUCUGC
		AGAACUUAGCCACUGUGAAUU
Hsa-miR-155-5p mimics	5’ to 3’	UUAAUGCUAAUCGUGAUAGGGGUU
		CCCCUAUCACGAUUAGCAUUAAUU
Hsa-miR-92a-3p mimics	5’ to 3’	UAUUGCACUUGUCCCGGCCUGU
		AGGCCGGGACAAGUGCAAUAUU
Hsa-miR-197-3p mimics	5’ to 3’	UUCACCACCUUCUCCACCCAGC
		UGGGUGGAGAAGGUGGUGAAUU
Negative control inhibitor	5’ to 3’	CAGUACUUUUGUGUAGUACAA
Hsa-miR-197-3p inhibitor	5’ to 3’	GCUGGGUGGAGAAGGUGGUGAA

Table S2. Quantitative reverse transcription polymerase chain reaction primers.

Gene		Sequence
CAMK2N1	Forward primer	GACACCAACAACTTCGGC
	Reverse primer	TCATCTTCAATAACAACCCGCTT
YTHDF3	Forward primer	GGTGTATTTAGTCAACCTGGGG
	Reverse primer	AAGAGAACTAGGTGGATAGCCAT
SLC2A12	Forward primer	AACATGCGGACCCGAATAATG
	Reverse primer	AATGACCTTGACGACTCCAAC
UGDH	Forward primer	CCCTGTGTGCTGTATATGAGC
	Reverse primer	TGCTTATTCTCTGGGCAAGAAAA
FKBP5	Forward primer	CTCCCTAAAATTCCCTCGAATGC
	Reverse primer	CCCTCTCCTTTCCGTTTGGTT
VDAC1	Forward primer	CTGACCTTCGATTCATCCTTCTC
	Reverse primer	CTCCCGCTTGTACCCTGTC
TXNDC5	Forward primer	CAGAGCCGGAAGTGGAACC
	Reverse primer	CCACGGAGCGAAGAACTTGAT
UHMK1	Forward primer	ACGCTGTCTGTTGCTTGAACT
	Reverse primer	GGCACAATGCTTGATCATCCAC
PARP2	Forward primer	TGCCCAGAGGAACTTCAGTG
	Reverse primer	TTGGTGGCATAGTCCATCTGT
GAPDH	Forward primer	ATACCAGGAAATGAGCTTGACAAA
	Reverse primer	GACATCAAGAAGGTGGTGAAGGAG
miR-197-3p	Special primer	TTCACCACCTTCTCCACCC

Figure S1. miR-197-3p suppresses colony formation in 22Rv1 cells.

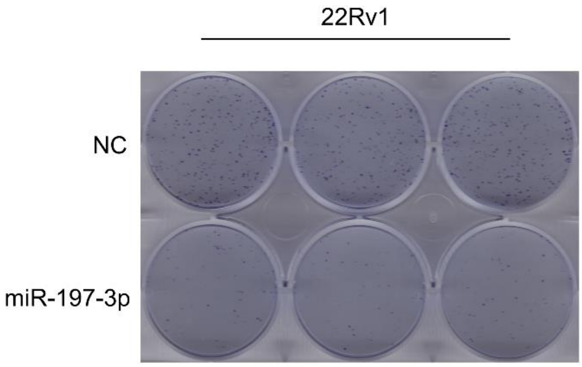

Figure S2. miR-197-3p has no effect on PCa cell migration and invasion.

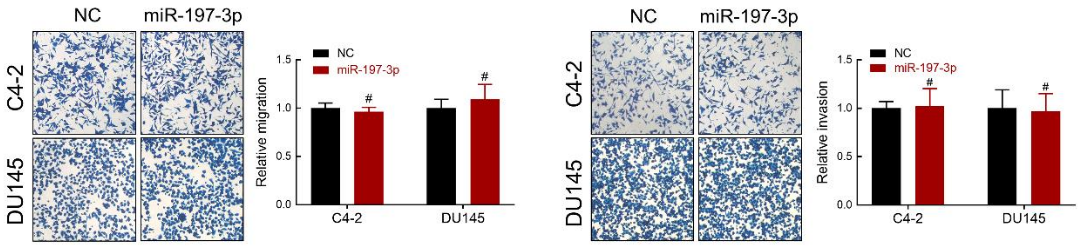

Figure S3. qRT-PCR was performed to confirm the treatment efficiency of miR-197-3p mimics.

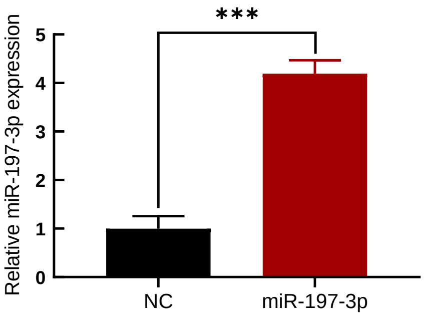

Supplement: Supplementary file 1 — Supplementary figures and tables. [file ijbsv16p1417s1.pdf]
